# Direct Induction of Chondrogenic Cells from Human Dermal Fibroblast Culture by Defined Factors

**DOI:** 10.1371/journal.pone.0077365

**Published:** 2013-10-16

**Authors:** Hidetatsu Outani, Minoru Okada, Akihiro Yamashita, Kanako Nakagawa, Hideki Yoshikawa, Noriyuki Tsumaki

**Affiliations:** 1 Department of Cell Growth and Differentiation, Center for iPS Cell Research and Application, Kyoto University, Kyoto, Japan; 2 Department of Orthopaedic Surgery, Osaka University Graduate School of Medicine, Suita, Osaka, Japan; 3 Japan Science and Technology Agency, CREST, Tokyo, Japan; University of Sao Paulo - USP, Brazil

## Abstract

The repair of large cartilage defects with hyaline cartilage continues to be a challenging clinical issue. We recently reported that the forced expression of two reprogramming factors (c-Myc and Klf4) and one chondrogenic factor (SOX9) can induce chondrogenic cells from mouse dermal fibroblast culture without going through a pluripotent state. We here generated induced chondrogenic (iChon) cells from human dermal fibroblast (HDF) culture with the same factors. We developed a chondrocyte-specific *COL11A2* promoter/enhancer lentiviral reporter vector to select iChon cells. The human iChon cells expressed marker genes for chondrocytes but not fibroblasts, and were derived from non-chondrogenic *COL11A2*-negative cells. The human iChon cells formed cartilage but not tumors in nude mice. This approach could lead to the preparation of cartilage directly from skin in human, without going through pluripotent stem cells.

## Introduction

Articular cartilage provides shock absorption and lubrication in diarthrodial joints. Articular cartilage is a hyaline cartilage which consists of chondrocytes and cartilage extracellular matrix composed of types II, IX and XI collagen molecules, proteoglycans, and other matrix proteins. Because hyaline cartilage has a poor intrinsic capacity for healing, the loss of cartilage due to trauma or degeneration caused by aging can result in debilitating conditions and osteoarthritis.

Cartilage damage sometimes heals with fibrocartilage, which differs from hyaline cartilage. Fibrocartilage is a type of scar tissue that expresses types I and II collagen; hyaline cartilage, in contrast, does not express type I collagen [1], [2]. As the presence of type I collagen impairs the development of cartilage-specific matrix architecture and mechanical function, the repair of cartilage damage by fibrocartilage results in morbidity and functional impairment. Therefore, the goal for repair of cartilage injury is the regeneration of organized hyaline cartilage, rather than healing with fibrocartilage [3]. Although autologous chondrocyte implantation has been successfully performed, the indications are limited to a small size defect, because chondrocytes are limited in number, and because they de-differentiate into fibrochondrocytes during monolayer expansion in culture [4]. Defects larger than 4 cm^2^ in size cannot be repaired because of the lack of a sufficient number of bona fide chondrocytes to fill the defect.

Sox9, Sox5 and Sox6 play important roles in the commitment of mesenchymal cells to the chondrocyte lineage. In mouse chimeras, *Sox9* ^−/−^ cells are excluded from the cartilage primordia throughout embryonic development [5], [6]. Sox9, Sox5, and Sox6 activate the transcription of chondrocyte-marker genes by binding their enhancers [7]–[10]. It was reported that the forced expression of Sox5, Sox6 and Sox9 by adenoviral vectors in dermal fibroblasts causes the expression of their target gene, type II collagen [11]. However, the histology of pellet cultures of these cells appears to be fibrocartilaginous, suggesting that the fibroblastic characteristics of the cells persist. Therefore, the cells produced in that study may correspond to fibrocartilaginous cells, rather than hyaline cartilaginous cells. Although dermal fibroblasts represent a readily accessible cell source, their tendency for high expression of type I collagen is a large obstacle to the production of hyaline cartilage. To eliminate the fibroblastic characteristics of these cells, a cell reprogramming process may be necessary.

A large number of autologous hyaline chondrogenic cells may be obtained by generating iPS cells, followed by redifferentiation into a chondrocytic lineage in the future. However, transplantation of redifferentiated chondrocytes is associated with a risk of teratoma formation due to the possible presence of residual undifferentiated cells. Recently, we induced chondrogenic cells directly from adult mouse dermal fibroblast (MDF) culture by transduction of two reprogramming factors (c-Myc, Klf4) and SOX9 [12]. The induced chondrogenic cells formed histologically homogenous hyaline cartilage when injected into the subcutaneous spaces of nude mice. Time-lapse observations of MDF cultures prepared from Nanog-GFP transgenic mice [13] revealed that GFP was not expressed during the induction of chondrogenic cells by transduction of c-Myc, Klf4, and SOX9, proving that the cells do not transition through a pluripotent state during the direct induction of chondrogenic cells from MDFs [14]. Therefore, the induced chondrogenic cells produced by this method are theoretically free from the risk of teratoma formation.

Future clinical application of this technique of regenerative medicine for articular cartilage diseases will require confirmation that chondrogenic cells can be directly induced from human dermal fibroblast culture. We here generated induced chondrogenic (iChon) cells from human dermal fibroblast (HDF) cultures. We employed nucleofection to misexpress Slc7a1, a receptor for retrovirus, followed by retroviral transduction of the same three factors, to cause the transformation of human dermal fibroblasts into chondrogenic lineage cells. We also developed a chondrocyte-specific *COL11A2* promoter/enhancer lentiviral reporter vector, to select human iChon cells. The human iChon cells expressed type II collagen, but not type I collagen. These human iChon cells generated stable homogenous hyaline cartilage-like tissue without tumor formation for at least 3 months in the subcutaneous spaces of nude mice.

## Materials and Methods

### Ethics Statement

All experiments were approved by our institutional animal committees, institutional biosafety committees, and institutional review boards of Osaka University and Kyoto University.

### Lentiviral Vectors and Transduction

The pLenti6/UbC/mSlc7a1 (Addgene plasmid 17224) was a gift from S. Yamanaka (Center for iPS Cell Research and Application (CiRA), Kyoto University, Kyoto, Japan) [15].

For construction of chondrocyte-specific reporter vectors, the human sequences corresponding to the mouse *Col11a2* promoter and enhancer [16] were amplified by PCR. The human *COL11A2* enhancer was linked to the EGFP-IRES-Puro sequence in the pENTR5′ plasmid (Invitrogen) [12] to prepare pENTR1A-mcs/(EGFP-IresPuro-hInt) (P4-40). The human *COL11A2* promoter was cloned into the pENTR5′ plasmid (Invitrogen) to prepare pENTR5′-mcs/11P (P4-41). The lentiviral vector, pLe6Δ (P4-32) was prepared by deleting the PGKpromoter-EM7-Blastcidine sequence at KpnI sites from pLenti6.4/R4R2/V5-DEST (Invitrogen). pENTR1A-mcs/(EGFP-IresPuro-hInt) (P4-40) was recombined with pENTR5′-mcs/11P (P4-41) and pLe6Δ by the LR clonase II plus reaction (Invitrogen) to prepare pLe6Δ -hLP-mcs/(EGFP-IresPuro-hInt) (P4-42, *COL11a2*-reporter vector), respectively. Lentiviral transduction was performed following the manufacturer’s instructions (Invitrogen).

### Cell Culture

Human dermal fibroblasts (HDFs) prepared from neonatal foreskin were purchased from Kurabo (KF-4009). On arrival, the HDFs were thawed, and 5×10^5^ cell were plated in a 10 cm dish in DMEM supplemented with 10% FBS. The next day, the cells were either untransduced, or were transduced with lentiviral *COL11A2*-reporter vectors overnight. The cells were subsequently split 1∶5 into 10 cm dishes and stored in liquid nitrogen until use. Human chondrosarcoma (HCS-2/8) cells were cultured in DMEM supplemented with 10% FBS [17].

### Chondrogenically Differentiated Human Bone Marrow Stem Cells

Human bone marrow stem cells (hBMSC) were purchased from Takara (PT-2501). On arrival of frozen stock of hBMSC, the cells were thawed and 3×10^5^ cell were plated in a 10 cm dish in αMEM supplemented with 10% FBS. When the cells became confluent, the culture was passaged (P1). Expanded hBMSC (P2) were suspended at 3×10^5^ cells/ml in αMEM containing 10% FBS, transferred into a 15-ml tube (Falcon), and centrifuged at 500 g for 5 min. The resulting cell pellet was incubated in a chondrogenic differentiation medium (purchased from Takara [PT-3003, PT-4124]) for 3 weeks, and the resulting cells were called chondrogenically differentiated human bone marrow stem cells (CD-hBMSC).

### Nucleofection of *Slc7a1*

The *Slc7a1* sequence from pLenti6/UbC/mSlc7a1 (Addgene plasmid 17224) was cloned into pDONR221 (Invitrogen) by BP clonase (Invitrogen) to prepare pDONR221-mSlc7a1 (P8-63). pDONR221-mSlc7a1 (P8-63) was recombined with pCMVb-gw (P1-32) by the LR reaction (Invitrogen) to prepare pCMV-gw/mSlc7a1 (P9-75). pCMV-gw/mSlc7a1 (P9-75) was introduced into HDFs using nucleofection technology following the manufacturer’s instructions (Amaxa).

### Retroviral Vectors and Transduction

pMXs-c-MYC (Addgene plasmid 17220) and pMXs-KLF4 (Addgene plasmid 17219), were gifts from S. Yamanaka (Center for iPS Cell Research and Application (CiRA), Kyoto University, Kyoto, Japan) [15]. pMXs-hSOX9 was described previously [12]. Human SOX5 and SOX6 cDNAs were PCR amplified using specific primers (Table S3) and were cloned into pDONR222 vector (Invitrogen) to create pENTR-hSOX5 (P5-41) and pENTR-hSOX6 (P5-42). pENTR-hSOX5 (P5-41) or pENTR-hSOX6 (P5-42) were recombined with pMXs-gw by the LR reaction (Invitrogen) to prepare pMXs-gw/hSOX5 (P8-83) or pMXs-gw/hSOX5 (P8-84). A sequencing analysis showed the hSOX5 and hSOX6 sequences to be correct.

Retroviral transduction was performed as described previously [18]. The Plat-E cells were a gift from T. Kitamura (The Institute of Medical Science, The University of Tokyo, Tokyo, Japan) [19].

Equal amounts of supernatants containing each of the retroviruses were mixed and added to the HDF cultures. After a 16-h incubation in the virus-containing medium, each fibroblast culture in the 10 cm dishes was trypsinized and split 1∶5 into new 10 cm dishes in fresh medium (DMEM supplemented with 10% FBS). The medium was changed every other day. In the cultures transduced with lentiviral *COL11A2*-reporter vectors, puromycin was added to the medium when GFP fluorescence was detected in some cells at around 7 days after retroviral transduction. At 21 days after retroviral transduction, dishes were subjected to alcian blue staining. The colony numbers were counted using the NIS Element software program (Nikon). We defined a colony as a cell cluster that was more than 0.5 mm in diameter. Other dishes were used for picking up colonies to generate induced chondrogenic cells.

### Alcian Blue Staining

Cells were fixed with methanol at −20°C for 2 min, incubated with 0.1% alcian blue (Sigma) in 0.1 N HCl for 2 h at 25°C, and washed three times with distilled water.

### Generation of Induced Chondrogenic Cell Lines

After setting up a sterile cylinder surrounding each colony, we harvested the cells by trypsinization, and replated them in 48 well dishes. After 6–10 days, the cells were replated in 24 well dishes. Then, cells were replated successively into 12 well, 6 well, 6 cm, and 10 cm dishes after culturing for them 3–14 days in each dish. We defined the stage of cells in 10 cm dishes as passage 6. The induced chondrogenic cells were cultured in DMEM containing 10% FBS in the presence of 1 µg/ml puromycin. Induced chondrogenic cells were passaged every 6 days. We initiated the growth curve analyses of induced chondrogenic cells at passage 11.

### Genomic PCR Analysis

The iChon cells (passage 11) and HDFs (passage 3) were cultured in 60 mm dishes. After the cells reached confluence, the genomic DNA was extracted and subjected to PCR to amplify transgenes. For the control, a fragment of the GAPDH gene was amplified. The primers used are listed in Table S2.

### RT-PCR and Real-time RT-PCR Analyses

After the iChon cells and HDFs cultured in 60 mm dishes reached confluence, the total RNA was extracted using RNeasy Mini Kits (Qiagen). The total RNAs prepared from the redifferentiated human fetal chondrocytes (HFC) were purchased from Cell Applications, Inc. (402RD-R10f). The total RNA was extracted from CD-hBMSCs. The total RNAs were digested with DNase to eliminate any contaminating genomic DNA. For RT-PCR analysis, 1 µg of total RNA was reverse transcribed into first-strand cDNA by using Superscript III Reverse Transcription (Invitrogen). PCR was performed with ExTaq (Takara). The primers used are listed in Tables S2 and S3. For real-time quantitative RT-PCR, 1 µg of total RNA was reverse transcribed into first-strand cDNA by using SuperScript III (Invitrogen) and an oligo(dT)_20_ primer. The PCR amplification occurred in a reaction volume of 20 µl containing 2 µl of cDNA, 10 µl of SYBER PremixExTaq (Takara) and 7900HT (Applied Biosystems). The PCR primers used are listed in Table S3. The RNA expression levels were normalized to the level of *GAPDH* expression.

### Determination of Karyotypes

iChon cells were subjected to karyotype analyses at Nihon Gene Laboratories (Japan).

### Immunofluorescence Staining

The cells were cultured on culture slides, fixed in 4% paraformaldehyde and permeabilized with 0.5% Tween 20. The cells were then incubated with the primary antibodies listed in Supplemental Table S4. Immune complexes were detected by using the appropriate secondary antibodies conjugated to Alexa Fluor (Table S4).

### Bisulfite Genomic Sequencing

Bisulfite treatment was performed by using the EpiTect Bisulfite kit (Qiagen) according to the manufacturer’s instructions. The PCR primers used are listed in Table S3. Amplified products were cloned into the pMD20-T vector using a Mighty TA-cloning Kit (Takara). Twelve randomly selected clones were sequenced with the M13 primer, RV, and M13 primer, M4, for each gene.

### Pellet Culture

Induced cells were suspended at 5×10^5^ cells/ml in DMEM containing 10% FBS, transferred into a 15-ml tube (Falcon), and centrifuged at 500 g for 5 min. The resulting cell pellet was incubated in chondrogenic medium (DMEM, 10% FBS, TGF-β 10ng/ml, DEX 10^−7^ M, Ascorbic acid 50 µg/ml, Pyrubate 100 µg/ml and ITS 6.25 µg/ml) for 3 weeks.

### Picrosirius Red Staining and Immunohistochemical Staining

Semi-serial histological sections were stained with picrosirius red using the Picrosisius red stain kit (Plysciences, Inc.) and immunostained with the primary and secondary antibodies listed in Table S4. For controls, sections from osteochodromas obtained at the time of surgery were used to test the anti-type I and anti-type II collagen antibodies.

### In vivo Cartilaginous Tissue Formation in Nude Mice

The iChon cells were suspended at 1×10^7^ cells/ml in DMEM containing 10% FBS. Then, 100 µl of the cell suspension was injected subcutaneously into the dorsal flank of 6-week-old female nude mice (BALB/cA Jcl-nu/nu). No carrier was used. The mice were sacrificed after 4, 8, 12 weeks, and the injected sites were dissected from the mice. Samples were fixed in 10% neutral buffered formalin, processed, and embedded in paraffin.

### Implantation of Human iChon Cells into the Articular Cartilage Defects in Severe Combined Immunodeficiency (SCID) Rats

The skin and joint capsule of a knee joint in 6-week-old SCID rats (F344-Il2rgtm2kyo) [20] were opened. A drill hole with a diameter of 1 mm was created at the femoral groove. A pellet of 5×10^5^ iChon cells was implanted into the hole, then the joint capsule and skin were closed. The rats were sacrificed four weeks later.

### Culture of iChon Cells Under Osteogenic Conditions

A total of 2×10^5^ iChon cells were plated in each well of a six well plate, and cultured in the osteogenic medium (α-MEM supplemented with 10% FBS, 10 mM β-glycerophosphate, 50 µg/ml ascorbic acid, and 10^−7^ M Dexamethasone in the absence or presence of various concentrations of BMP2). The medium was changed every other day. RNAs were extracted from the cells after 21 days of culture, and were subjected to a real-time RT-PCR expression analysis. As control, RNAs were extracted from subchondral bone samples collected from the tibia and femur at the time of surgery.

### Implantation of iChon Cells into the Calvarial Defects of SCID Mice

The skin on the head of 5-week-old SCID mice (C.B-17/lcr-scid/scid Jcl) was incised, then a drill hole with a diameter of 1 mm was created at the calvarium. A pellet of 5×10^5^ iChon cells was implanted into the hole, then the skin was closed. The mice were sacrificed three weeks later.

### Statistical Analysis

The data are shown as averages and standard deviations. Two-tailed Student’s *t*-tests were used to compare the data. P values <0.05 were considered to be statistically significant.

## Results

### The Generation of Human Induced Chondrogenic (iChon) Cells from HDFs by Transduction of c-MYC, KLF4 and SOX9

**Figure 1:**
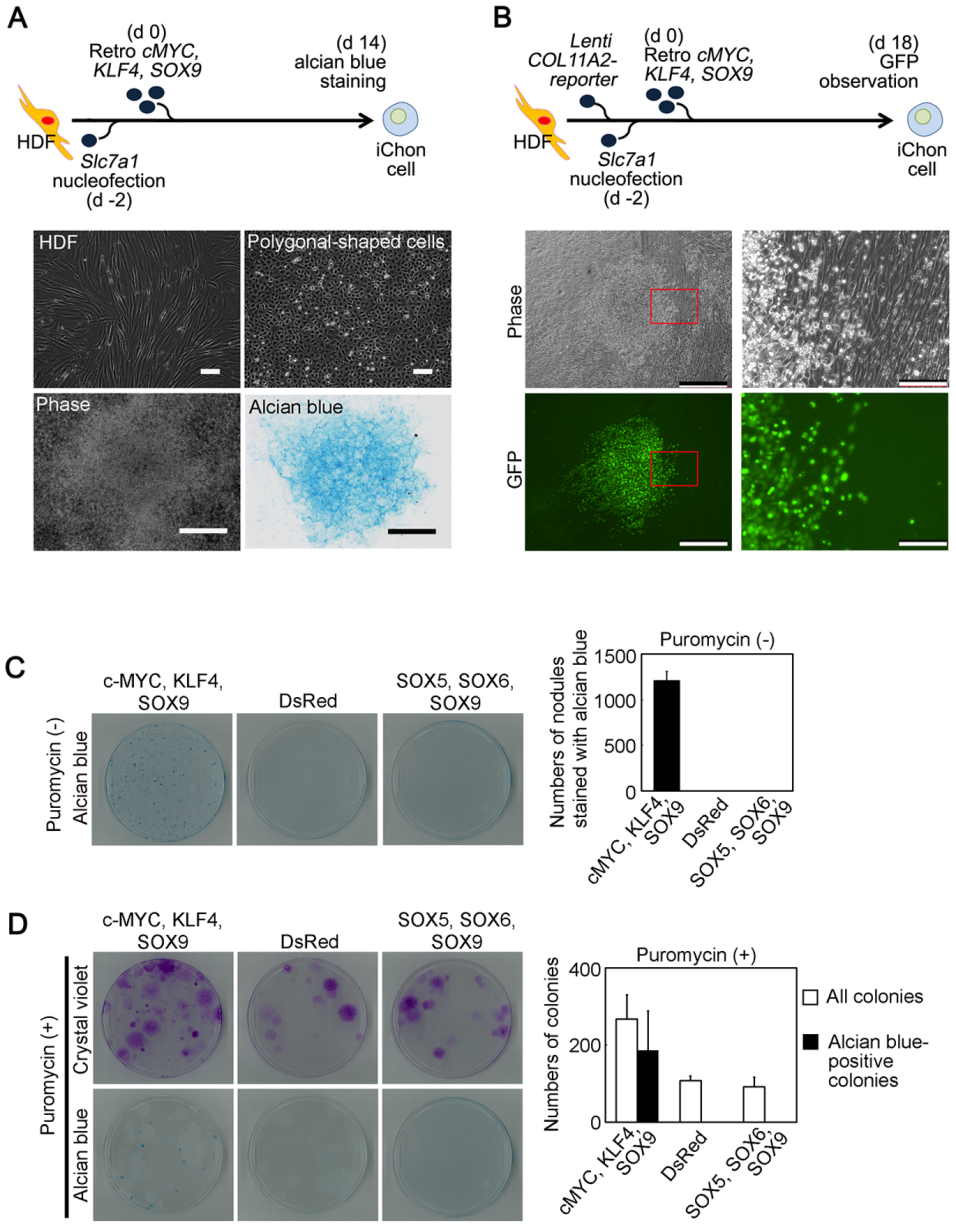
The generation and selection of induced chondrogenic (iChon) cells from human dermal fibroblast (HDF) culture. (**A**) Top, a schematic diagram of the gene transduction. Middle left, HDFs. Middle right, polygonal-shaped cells generated by transduction of c-MYC, KLF4, and SOX9. Bottom left, nodules formed by polygonal-shaped cells. Bottom right, nodules were intensely stained with alcian blue, suggesting the production of glycosaminoglycan. Bars in top panels, 100 µm; Bars in bottom panels, 500 µm. (**B**) Top, a schematic diagram of the gene transduction. Left middle and bottom panels, phase and GFP images of cell nodules formed in HDF culture at 18 days after transduction with c-MYC, KLF4 and SOX9. Right middle and bottom panels, magnification of the boxed region in the left panels. Cells were cultured in the absence of puromycin Bars in left panels, 500 µm; Bars in right panels, 100 µm. (**C**) HDF cultures which had been transduced with lentiviral *COL11A2-* reporter vectors and nucleofected with *Slc7a1* were transduced with retroviral c-MYC, KLF4 and SOX9, or DsRed fluorescent protein, or SOX5, SOX6 and SOX9. Cells were cultured in the absence of puromycin. Dishes (10 cm in diameter) were stained with alcian blue 21 days after retroviral transduction. The numbers of nodules with positive alcian blue staining were counted. (**D**) HDF cultures which had been transduced with lentiviral *COL11A2-* reporter vectors and nucleofected with *Slc7a1* were transduced with retroviral c-MYC, KLF4 and SOX9, or DsRed fluorescent protein, or SOX5, SOX6 and SOX9. Puromycin was added to the medium 7 days after retroviral transduction. Dishes (10 cm in diameter) were stained with crystal violet and alcian blue 21 days after retroviral transduction. The numbers of all colonies stained with crystal violet (white bars) and the numbers of colonies with positive alcian staining (black bars) were counted. In (C and D), after nucleofection of *Slc7a1*, HDFs were replated at a density 5×10^5^ cells per 10 cm dish for retroviral transduction. Cells were split 1∶5 onto 10 cm dishes immediately after completion of the retroviral transductions. The numbers of nodules in five 10 cm dishes which were derived from one identical dish were added together. Error bars indicate ± SD (*n* = 3 dishes).

We first transduced neonatal foreskin HDF cells (Figure 1A, middle left panel) with lentiviral vectors bearing human c-MYC, KLF4 and SOX9, but failed to obtain cells with the polygonal morphology which is typical shape of cultured chondrocytes. Therefore, we transduced HDFs with these factors following another previously described method using *Slc7a1* encoding a receptor for the retrovirus [15]. We tried to increase the titer of retroviral infection by transducing HDFs with *Slc7a1* using nucleofection technology to increase the expression levels of *Slc7a1*. Two days after the nucleofection of *Slc7a1*, we transduced the HDFs with retroviral c-MYC, KLF4 and SOX9 vectors. Five days after retroviral transduction, we detected polygonal cells in the HDF culture. The polygonal cells (Figure 1A, middle right panel) formed multiple layers at 14 days after transduction, giving rise to cell nodules (Figure 1A, bottom left panel) which is a characteristic of cultured primary chondrocytes [21]. The nodules were surrounded by cells that had the morphological appearance of fibroblasts. These nodules were specifically and intensely stained with alcian blue (Figure 1A, bottom right), suggesting the existence of acid glycosaminoglycans, which is an element of cartilage extracellular matrix. The surrounding cells that had the morphological appearance of fibroblasts were not stained with alcian blue. These results suggest that forced expression of c-MYC, KLF4 and SOX9 can produce induced chondrogenic (iChon) cells from human skin fibroblast cultures.

### Selection of Human iChon Cells with a *COL11A2* Enhancer-based Lentiviral Reporter Vector

To remove fibroblastic cells and isolate homogenous polygonal-shaped iChon cell populations, we constructed a chondrocyte-specific reporter vector using EGFP and puromycin-resistant genes linked by the IRES sequence on the lentiviral vector. To obtain chondrocyte-specific expression, we employed promoter and enhancer sequences of the human α2(XI) collagen chain (*COL11A2*) gene (Figure S1A) that correspond to the mouse *Col11a2* promoter and enhancer, which direct chondrocyte-specific expression [16]. The *COL11A2-*reporter vector directed substantial GFP expression in human chondrosarcoma (HCS-2/8) cells [17] but not in HDFs (Figure S1B). We transduced the HDFs with lentiviral *COL11A2-*reporter vectors overnight, split them 1∶5, and made a cell stock from these cells. The cell stock of HDFs transduced with reporters was thawed (day 0) and we nucleofected the cells with *Slc7a1* (day 2). We then replated 5×10^5^ cells in a 10 cm dish (day 3) and transduced the cells with retroviral vectors bearing c-MYC, KLF4 and SOX9 (day 5) overnight. Immediately after retroviral transduction, the cells were split 1∶5 in 10 cm dishes (day 6) (Figure 1B). Polygonal-shaped cells, which appeared 5 days after retroviral transduction with c-MYC, KLF4 and SOX9, started to show *COL11A2-GFP* fluorescence. Subsequently, the polygonal-shaped cells formed nodules. EGFP was expressed in the nodules, but not in the surrounding fibroblastic cells in the absence of puromycin (Figure 1B). The transduction of 5×10^5^ HDFs with c-MYC, KLF4 and SOX9 resulted in the formation of 1200 nodules, which showed intense staining for alcian blue in the absence of puromycin (Figure 1C) in five 10 cm dishes. Therefore, the efficiency of the induction of alcian blue-positive cells was 0.24%. On the other hand, the transduction of HDFs with the control retroviral DsRed fluorescent protein vector resulted in neither nodule formation nor substantial alcian blue staining (Figure 1C). Because a combination of SOX9, SOX5 and SOX6 was previously reported to activate the expression of chondrocyte-markers in HDFs [11], we transduced HDFs with SOX9, SOX5 and SOX6. We confirmed that the SOX5, SOX6 and SOX9 proteins were expressed by immunoblot analyses (Figure S2). We found neither nodule formation nor any substantial alcian blue staining in the HDFs transduced with SOX9, SOX5 and SOX6 (Figure 1C). These results indicate that c-MYC and KLF4 play a critical role in the conversion from fibroblasts to chondrogenic cells by SOX9.

To isolate colonies, we started to add puromycin to the medium when GFP fluorescence was detected at 5–7 days after transduction with c-MYC, KLF4 and SOX9. The majority of fibroblastic cells and four-fifths of the polygonal-shaped cells died, leaving 267 surviving colonies, on average, in the culture in the presence of 1 µg/ml puromycin (Figure 1D). On average, 186 out of the 267 colonies were intensely stained with alcian blue and were composed of polygonal cells. The remaining 81 colonies were large and diffuse, as indicated by crystal violet staining, and were composed of fibroblastic cells. The transduction of HDFs with the control retroviral DsRed vector instead of c-MYC, KLF4 and SOX9 vectors produced 107 colonies in the presence of puromycin. These colonies were not stained with alcian blue (Figure 1D), and were composed of fibroblasts. These fibroblasts survived in the presence of puromycin, probably because of the aberrant expression of the lentiviral *COL11A2*-reporter vector. The transduction of HDFs with SOX9, SOX5 and SOX6 also produced 91 colonies in the presence of puromycin. These colonies did not stain for alcian blue (Figure 1D), and were composed of morphologically fibroblastic cells.

Therefore, the transduction of the lentiviral *COL11A2* reporter vector and selection with puromycin resulted in the formation of nodules as colonies, 70% of which were composed of polygonal-shaped cells. The colonies composed of polygonal cells were almost all intensely stained with alcian blue. We picked up colonies which were composed of polygonal-shaped cells, and added them in the wells of 48 well plates and expanded each cell line by replating them onto successively larger dishes. We established 15 clones which reached subconfluency in 10 cm dishes, and were subjected to the further analyses.

### Characteristics of the iChon Cells

**Figure 2:**
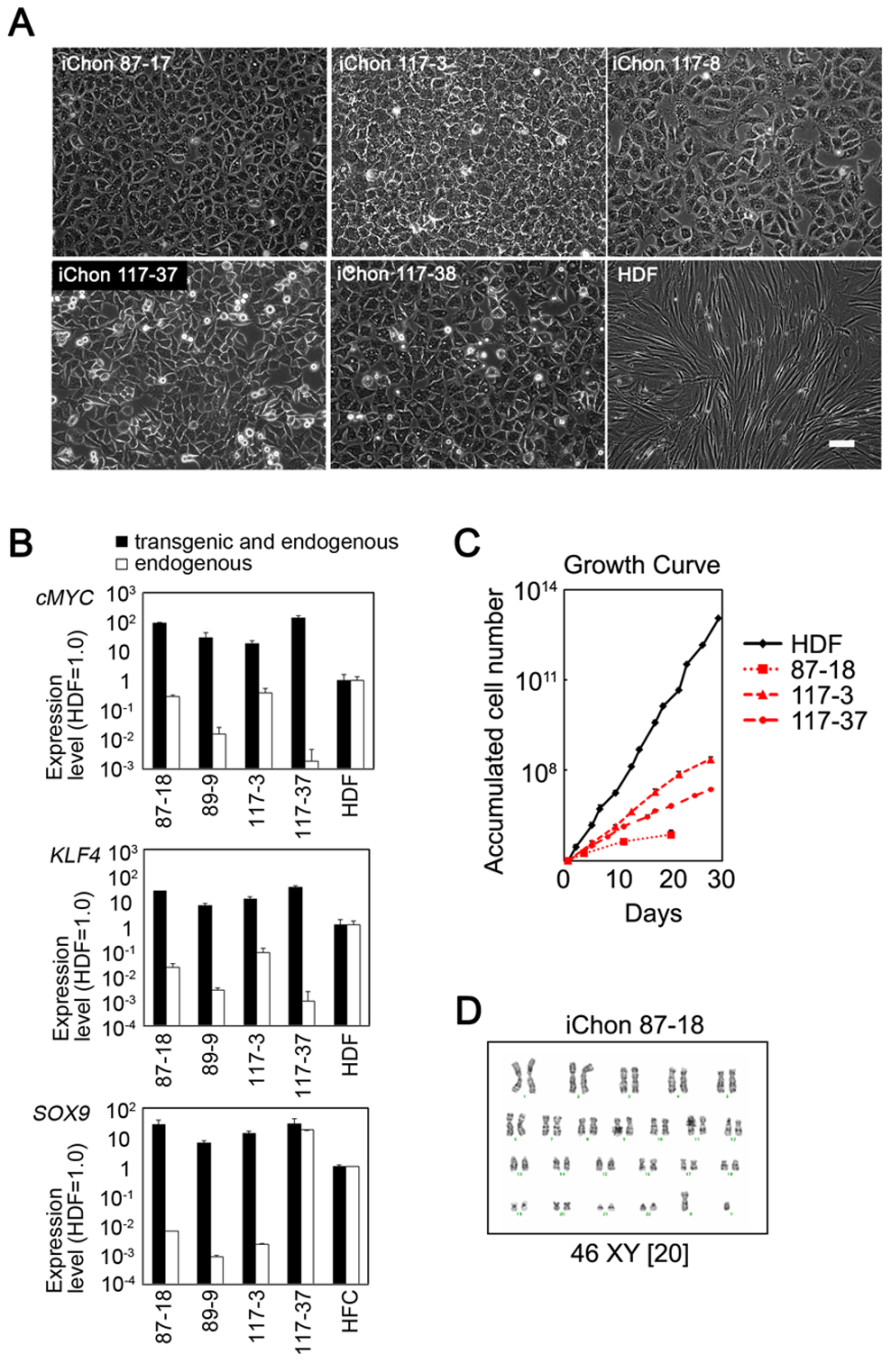
The characteristics of human iChon cell lines. (**A**) Phase images of human iChon cell lines and HDFs. The photos were taken when the cell numbers had expanded and reached 10^7^. Bar: 100 µm. (**B**) The mRNA levels of *cMYC*, *KLF4* and *SOX9* in iChon cells. The relative expression levels in comparison to human HDFs or HFCs are shown. The mRNA levels were determined by a real-time RT-PCR analysis using primers specific for endogenous transcripts (white columns) and those common for both transgenic and endogenous transcripts (black columns). The error bars indicate ± SD (*n* = 3). (**C**) The growth curves of human iChon cells and parental HDFs. (**D**) The karyotype of human iChon cells. iChon cell line #87-18 was examined at passages 15. A total of 20 cells for each cell line were examined. HDF, human dermal fibroblasts; HFC, redifferentiated human fetal chondrocytes.

Isolated human iChon cells showed a polygonal morphology (Figure 2A). We found that all three (c-MYC, KLF4 and SOX9) transgenes were transduced into the genomic DNA (Figure S3A) and were expressed (Figure 2B) in iChon cells. These results suggest that retrovial transgenes were not silenced in iChon cells. A growth curve analysis showed that the proliferation rates of the established human iChon cell lines were lower than those of the parental HDFs (Figure 2C). A karyotype analysis showed that 20 out of 20 cells (iChon 87-18), 20 out of 20 cells (iChon 117-3) and 14 out of 20 cells (iChon 117-37) had normal karyotypes (Figure 2D and Figure S3B).

**Figure 3:**
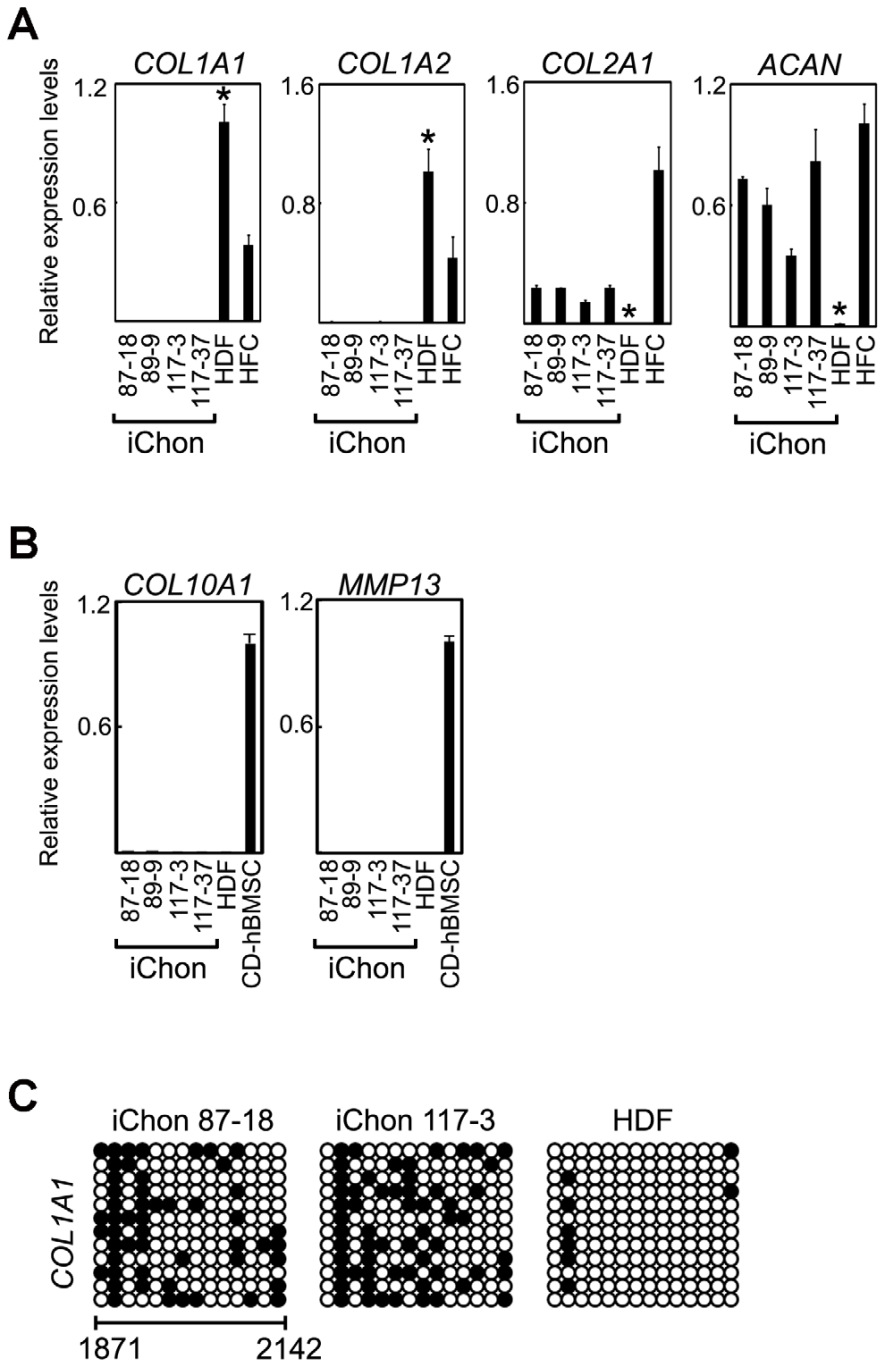
Marker gene expression of human iChon cell lines. RNA samples were extracted from iChon cells at passage 7. (**A**) The quantitative expression analyses of chondrocyte and fibroblast marker genes in human iChon cell lines, HDFs and HFC. Error bars indicate the ± SD (*n* = 3). *P<0.01 compared with iChon cell lines by Student’s t-test. (**B**) The quantitative expression analyses of chondrocyte hypertrophy and terminal differentiation marker genes in human iChon cell lines, HDFs and CD-hBMSCs. Error bars indicate the ± SD (*n* = 3). The relative expression levels of *COL10A1* and *MMP13* mRNAs were zero in iChon cell lines. (**C**) Methylation of the regulartory region of the *COL1A1* gene. Bisulfite genomic sequencing of the regulartory regions of *COL1A1* was performed using DNA derived from iChon cell lines and HDFs. Each horizontal row of circles represents an individual sequencing result from one amplicon. Open circles indicate unmethylated CpG dinucleotides, while closed circles indicate methylated CpGs. The nucleotide numbers for *COL1A1* are indicated at the bottom. The ATG translation initiation codon is set as +1 (GenBank accession number NC 000017, nt 48261457). HDFs, human dermal fibroblasts; HFC, redifferentiated human fetal chondrocytes; CD-hBMSCs, chondrogenically differentiated human bone marrow stem cells.

A real-time RT-PCR analysis showed that human iChon cells expressed neither *COL1A1* nor *COL1A2* mRNAs, which the parental HDFs expressed abundantly (Figure 3A). With regard to the control, redifferentiated human fetal chondrocytes (HFC) expressed both *COL1A1* and *COL1A2*, probably because fibroblasts had contaminated the cells during their collection, or because of dedifferentiation of chondrocytes during culture. On the other hand, the iChon cells expressed chondrocyte-specific marker genes, *COL2A1* and *ACAN*, whereas the HDFs did not. The iChon cell lines showed little expression levels of the hypertrophic chondrocyte marker *COL10A1* and terminally differentiated chondrocyte marker *MMP13* (Figure 3B). Regarding the control, we prepared chondrogenic cells by differentiation from human bone marrow stem cells. Chondrogenically differentiated human bone marrow stem cells (CD-hBMSCs) expressed *COL10A1* and *MMP13*. These expression patterns of iChon cells were maintained after passaging them (Figure S3C).

The bisulfate sequencing analysis revealed that the cytosine guanine (CpG) dinucleotides in the regulatory element of the fibroblast-marker gene *COL1A1* were demethylated in human iChon cells, whereas it was not methylated in the parental HDFs (Figure 3C). These results suggest that the fibroblast marker gene, *COL1A1,* was silenced during the induction of human iChon cells.

**Figure 4:**
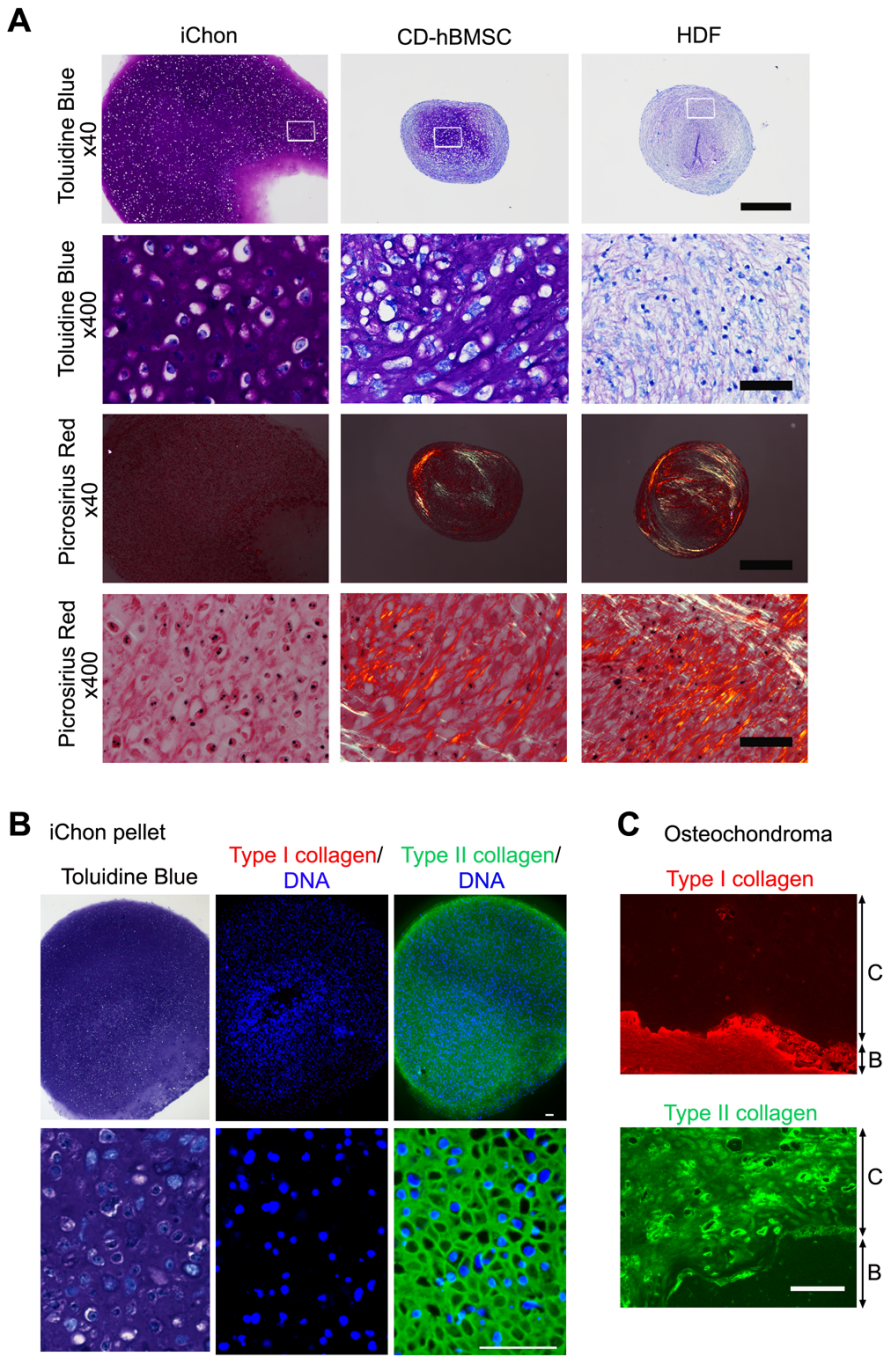
Characterization of matrix of pellet culture of iChon cells. The iChon cells at passage 7 were used. (**A**) After 3 weeks of culture, pellets of iChon cells (# 117-3), chondrogenically differentiated human bone bone marrow stem cells (CD-hBMSC), and human dermal fibroblasts (HDFs) were recovered and processed for histological sections. Semiserial sections were stained with toluidine blue and picrosirius red. Sections stained with picrosirius red were observed under polarized microscopy. Bars in the top and third rows, 500 µm; Bars in the second and bottom rows, 50 µm. (**B**) Semiserial sections of pellets of iChon cells (#117-37) after 3weeks of culture were stained with toluidine blue, and immunostained with anti-type I collagen antibodies and anti-type II collagen antibodies. Bars, 100 µm. (**C**) Control for immunohistological analysis in (B). Histological sections from osteochondroma samples dissected at a time of surgery were immunostained with anti-type I collagen and anti-type II collagen antibodies. Panels are magnification of boxed regions in Figure S7. Bar, 100 µm. *C*, cartilage; *B*, bone.

We next investigated the cartilage tissue-forming activities of iChon cells by pellet culture. The histology of the pellet culture of iChon cells showed cartilaginous structures (Figure 4A). The matrix of the iChon pellet showed intense metachromatic toluidine blue staining, whereas that of CD-hBMSC pellet looked fibrous. The matrix was hardly formed in the HDF pellet. Picrosirius red staining revealed the fibrous alignment of collagen fibers in CD-hBMSC pellet and HDF pellet, but not in the iChon pellet under polarized microscopic observations. These results suggest that the matrix of the iChon pellet has a hyaline cartilaginous structure; whereas the matrix of the CD-hBMSC pellet has a fibrocartilaginous structure. Immunohistochemistry showed that the matrix contained type II collagen, but not type I collagen (Figures 4B and 4C). These results suggest that human iChon cells produce hyaline cartilage *in vitro*.

### The Origin of Induced Chondrogenic Cells in the HDF Culture

**Figure 5:**
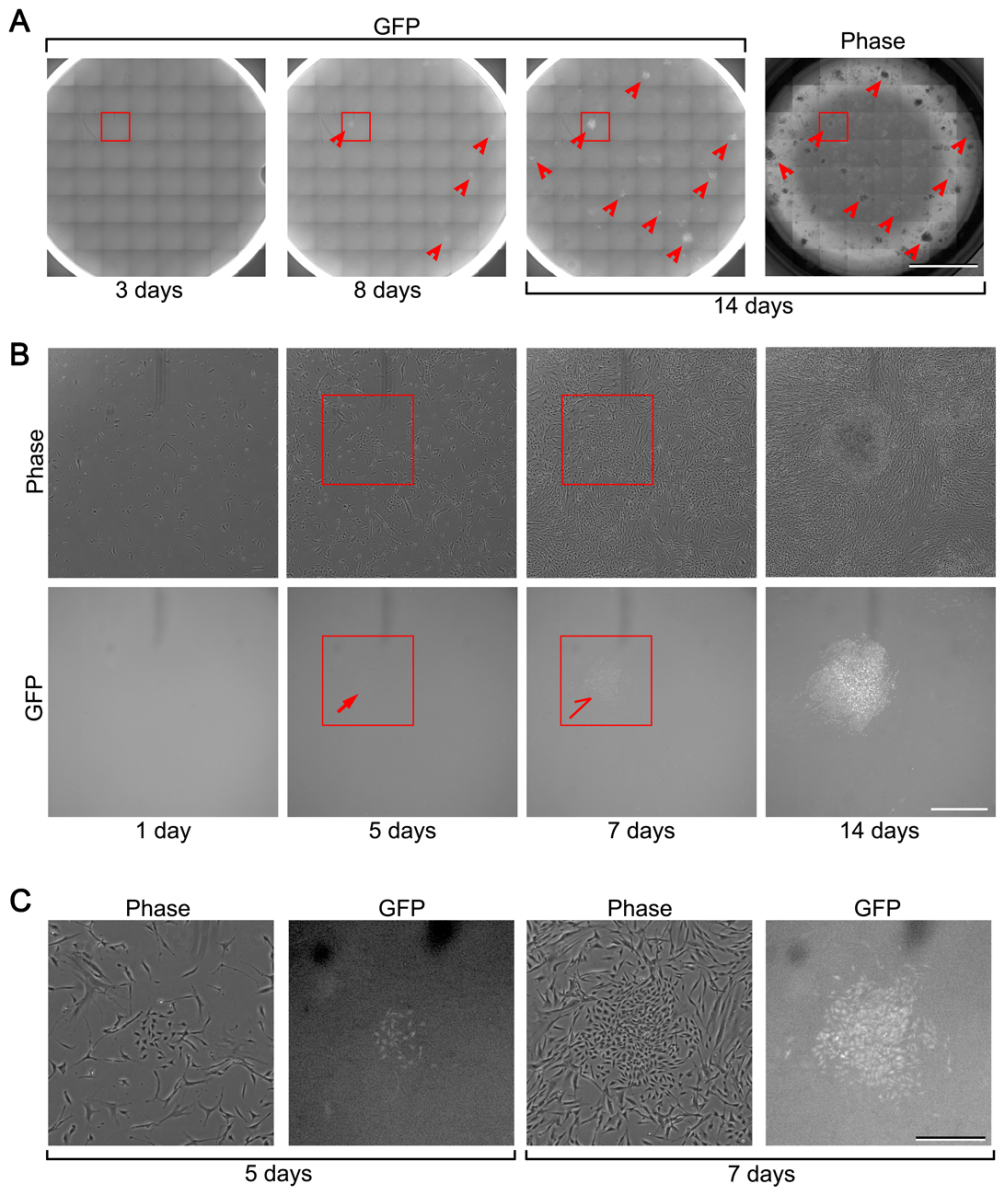
The origins of iChon cells in HDF culture. HDFs were transduced with the lentiviral *COL11A2*-reporter vector, nucleofected with Slc7a1, and transduced with retroviral c-MYC, KLF4 and SOX9 vectors. Cells were replated onto a well of a 6 well plate immediately after completion of the retroviral transduction. The well was cultured in the absence of puromycin and subjected to time-lapse GFP observation using the Biostation CT (Nikon). (**A**) The entire wells were each scanned using a total of 64 images (8 rows×8 columns), and a tiled image was reconstituted. The time-lapse GFP fluorescence of the tiled images at 3, 8 and 14 days after transduction of retroviral c-MYC, KLF4 and SOX9 vectors (3 left panels), and a phase contrast image 14 days after transduction (right panel), spanning an entire well of a 6 well plate are shown. GFP fluorescence was not observed at 3 days after retroviral transduction. Some cell clusters expressed *COL11A2-GFP* fluorescence at 8 days after transduction (arrowheads), and gradually formed nodules, increasing the level of GFP fluorescence. At 14 days after transduction, some nodules (arrowheads) specifically expressed *COL11A2-GFP* and others did not. Bar, 10 mm. (**B**) The magnification of the boxed regions in (A). At 1 day after transduction, no cells expressed *COL11A2-GFP*, suggesting that they were not chondrogenic cells. A cluster of cells with polygonal morphology started to express *COL11A2-GFP* weakly (arrow) at 5 days after transduction. The cells in the cluster increased in number and expressed *COL11A2-GFP* (half-arrow) at 7 days after transduction. A cell cluster formed multiple layers, forming a nodule which expressed *COL11A2-GFP* strongly at 14 days after transduction. These results suggest that iChon cells are derived from non-chondrogenic cells which did not express *COL11A2-GFP*. Bar, 100 µm. (**C**) Magnification of the boxed regions in (B). GFP images were enhanced to detect weak fluorescent signals. Only the polygonal cell clusters expressed *COL11A2-GFP*, but the surrounding fibroblast cells did not express *COL11A2-GFP*. Bar, 50 µm.

To gain insight into the original cell type which gives rise to chondrogenic cells in HDF culture, we performed time-lapse observations of whole wells of a 6-well plate during the induction of chondrogenic cells (Figure 5). *COL11A2-GFP* fluorescence was not observed at 3 days after retroviral c-MYC, KLF4 and SOX9 transduction throughout the whole wells (Figure 5A, left panel). Some cell clusters expressed *COL11A2-GFP* fluorescence at 8 days after transduction (Figure 5A, second panel, arrowheads), and gradually formed nodules, increasing the level of GFP fluorescence. At 14 days after transduction, some nodules (Figure 5A, third panel, arrowheads) specifically expressed *COL11A2-GFP,* while others did not. We retrospectively analyzed 23 nodules of chondrogenic cells which expressed *COL11A2-GFP* and identified their original cells at the start of induction. The cells of origin of all 23 nodules expressing *COL11A2-GFP* did not express GFP at the start of the induction. A close examination revealed that the cells from which the *COL11A2-GFP*-positive cells originated did not express *COL11A2-GFP* one day after transduction with c-MYC, KLF4 and SOX9, and only started to express *COL11A2-GFP* at 5–7 days after transduction (Figures 5B and 5C, and Movies S1 and S2). The mouse *Col11a2* regulatory sequences which correspond to the human *COL11A2* promoter-enhancer used in the lentiviral *COL11A2*-reporter gene direct the expression from prechondrogenic cells during mesenchymal condensation [12], [16].

In addition, we examined the proportions of cells expressing SOX9 in HDF culture. SOX9 is known to be expressed in chondroprogenitor cells [22]. Immunofluorescence staining of parental HDFs with anti-SOX9 antibodies revealed that the signals were at the background level in almost all cells (Figure S4). The ratio of possible prechondrogenic cells indicated by immunoreactivity against anti-SOX9 antibody in the HDF culture (0.019%, Figure S4C) was lower than the frequency of alcian blue-positive cells generated from HDFs (0.24%, Figure 1D). A close examination revealed that almost all SOX9-positive signals in the HDF culture were false-positive signals, because the signals were on the edge of cells or debris and did not localize in in the nucleus. These results collectively suggest that non-chondrogenic cells were the major source of the iChon cells.

### Hyaline Cartilage Formation by Human iChon Cells in the Subcutaneous Spaces in Nude Mice

**Figure 6:**
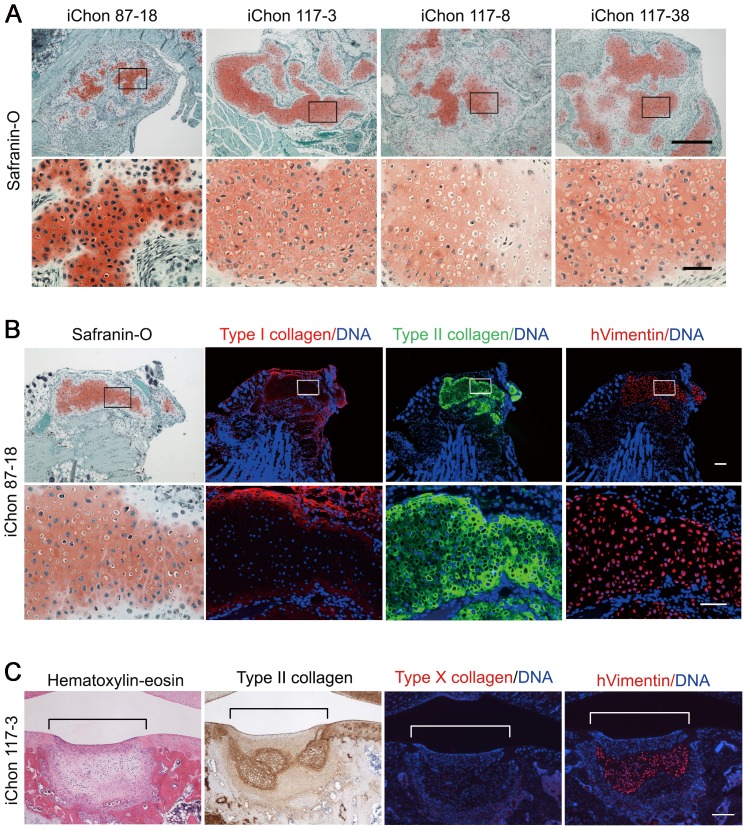
*In vivo* cartilage formation by human iChon cells in the subcutaneous spaces of nude mice (A and B) and articular cartilage defects created in SCID rat (C). The iChon cells at passage 7 were used. Mice were sacrificed at 4 weeks after subcutaneous injection of iChon cells, and nodules at the injected sites were collected. Rats were sacrificed 4 weeks after implantation. (**A**) The histological features of nodules formed by injected iChon cells into subcutaneous spaces of nude mice. Safranin O-fast green-iron hematoxylin staining. Cartilaginous matrix was specifically stained with Safranin O as an orange color. Bars in top panels, 500 µm; Bars in bottom panels, 100 µm. (**B**) The expression of differentiation-related proteins in nodules formed by injected iChon cells into subcutaneous spaces of nude mice. Semiserial sections of nodules derived from injected 87-18 human iChon cell were stained with Safranin O-fast green-iron hematoxylin and immunostained with anti-type I collagen, anti-type II collagen and anti-human vimentin antibodies. The bars in top panels, 500 µm; in bottom panels, 100 µm. (**C**) Human iChon cells (line #117-3) were implanted into defects created in the articular cartilage of the distal femurs of SCID rats. Four weeks after implantation the rats were sacrificed. Semiserial sections were stained with hematoxylin-eosin and immunostained with anti-type II collagen, anti-type X collagen, and anti-human vimentin antibodies. Brackets indicate regions of defects created in the articular cartilage. The bars, 100 µm.

We next investigated the cartilage-forming activities of iChon cells *in vivo*. We injected independent iChon cell lines suspended in the medium into the subcutaneous spaces of nude mice (Table S1). Four weeks after injection, we found solid nodules at 14 out of the 42 sites that were injected (33%). We found no nodule formation in the other 28 sites. A histological analysis revealed that these nodules contained cartilage-like tissue (Figure 6A). Cells were scattered in the matrix, which was positively stained with safranin O. We recognized that the cells resided in lacuna, which is characteristic of cartilage histology. An immunohistochemical analysis showed that the matrix contained type II collagen, but not type I collagen, suggesting that the tissue formed by the injection of human iChon cells was hyaline cartilage (Figure 6B). The cells in the cartilage expressed human vimentin, indicating that the injected iChon cells survived and formed cartilage. Longer-term observation showed that the formed cartilage gradually disappeared from the subcutaneous spaces (Table S1). We found no tumor formation at 76 injected sites, including 17 sites that were observed for 3 months (Table S1).

### Cartilaginous Tissue Formation by Human iChon Cells in the Articular Cartilage Defects Created in the SCID Rat

We further examined whether iChon cells form cartilage in orthotopic sites. We implanted human iChon cells into defects created in the articular cartilage of six knees of SCID rats. Four weeks after implantation, the defects were partially filled with cartilaginous tissue in four out of the six knees (Figure 6C). The cartilaginous tissues showed positive immunostaining for type II collagen. The expression of type X collagen was below the limit of detection. Immunostaining for human vimentin showed that the cartilaginous tissue was composed of human cells. The human iChon cell-derived cartilaginous tissue was surrounded by scar tissue which consisted of host cells. These results suggest that human iChon cells survive and form cartilaginous tissue in the articular cartilage defects for at least four weeks.

### The Low Susceptibility of Human iChon Cells to Osteogenic Conditions

**Figure 7:**
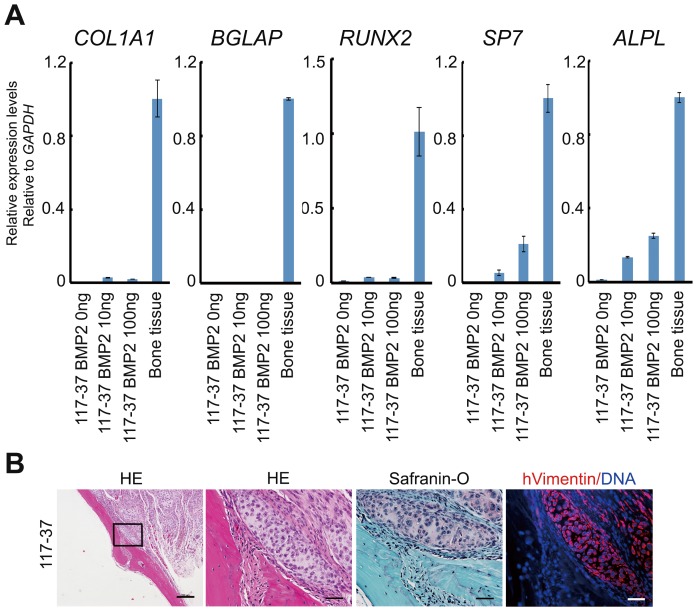
The response of human iChon cells to osteogenic conditions. (**A**) Human iChon cells (line #117-37) were cultured in the osteogenic medium (α-MEM supplemented with 10% FBS, 10 mM β-glycerophosphate, 50 µg/ml ascorbic acid, and 10^−7^ M dexamethasone with the absence or presence of various concentrations of BMP2 as indicated at the bottom of the graphs) for 21 days. RNAs were extracted and subjected to a real-time RT-PCR expression analysis. Error bars indicate the ± SD (*n* = 3). (**B**) Human iChon cells were implanted into defects created in the calvaria of SCID mice. Three weeks after implantation, the mice were sacrificed. Semiserial sections were stained with hematoxylin-eosin and safranin O, and immunostained with anti-human vimentin antibodies. The magnification of boxed regions in the left panel is shown in the right three panels, respectively. The bars in the left panels, 250 µm; in the other panels, 50 µm.

We examined how iChon cells respond to osteogenic conditions. Human iChon cells did not express the OSTEOCALCIN gene (*BGLAP*) or RUNX2 gene when they were cultured in the osteogenic medium containing 100 ng/ml BMP2 for 21 days, although expression of the OSTERIX gene (*SP7*) and the alkaline phosphatase gene (*ALPL*) were slightly increased by the addition of BMP2 (Figure 7A). The *SP7* and *ALPL* genes were expressed in chondrocytes as well as osteoblasts.

Human iChon cells produced cartilaginous tissue and did not form bone, when implanted into the defects created in the calvaria of SCID mice (Figure 7B). The calvarial defects were healed spontaneously, and found to be filled with host bone tissue. These results collectively suggest that human iChon cells did not respond to osteogenic conditions.

## Discussion

Although small articular cartilage defects measuring less than 2 cm^2^ in size can be treated with autologous chondrocyte transplantation, the treatment of larger cartilage defects remains a challenge. Cell reprogramming techniques have the potential to resolve this problem by providing a sufficient number of hyaline chondrocytes to fill large defects. In this study, we generated human iChon cells directly from HDF culture by transduction of two reprogramming factors (c-MYC and KLF4) and one chondrogenic factor (SOX9). Human iChon cells were generated from non-chondrogenic cells in HDFs, as indicated by the lack of *COL11A2* promoter/enhancer activities and the fact that the majority of the cells had no endogenous SOX9 expression. Human iChon cells generated hyaline cartilage without tumor formation in the subcutaneous space of nude mice. Human iChon cells also formed cartilage in the defects of articular cartilage and did not respond to osteogenic conditions. These results suggest that human iChon cells can be a candidate cell source for regenerative medicine to treat articular cartilage diseases.

It was previously reported that a high level of overexpression of SOX5, SOX6 and SOX9 could activate the expression of chondrocyte-markers in HDFs using adenoviral vectors [11], although fibroblastic characteristics appeared to be remained. We found that the retroviral transduction of c-MYC, KLF4, and SOX9 following *Slc7a1* nucleofection produced substantial numbers of cartilaginous nodules in HDF culture, whereas retroviral transduction of SOX5, SOX6 and SOX9 following *Slc7a1* nucleofection did not. These results indicate that the reprogramming factors c-MYC and KLF4 more efficiently contribute to the SOX9-induced conversion of fibroblasts into chondrogenic cells than SOX5 and SOX6. c-Myc and Klf4 are responsible for erasing the characteristics of fibroblasts during iPS cell induction by c-Myc, Klf4, Oct3/4 and Sox2 [23]. The expression of fibroblast markers was observed to decrease first, followed by an increase in the expression of chondrocyte markers during the induction of mouse chondrogenic cells from MDFs by c-Myc, Klf4 and SOX9 [14]. These findings suggest that c-MYC and KLF4 are involved in epigenetic events in HDFs, and enable SOX9 to direct cells to the chondrogenic lineage during the induction of iChon cells.

The c-MYC, KLF4, and SOX9 transgenes were not silenced in human iChon cells. The silencing of retroviral transgenes is a phenomenon that is a characteristic of pluripotent cells [24] including iPS cells [15]. Retroviral transgenes are not usually silenced in somatic cells and somatic cells which are produced by direct conversion technique [12], [25], [26]. Human iChon cells expressed neither type X collagen nor MMP13. Human iChon cells retain their chondrogenic phenotype after being passaged in monolayer culture. These results suggest that human iChon cells therefore stay in hyaline chondrocytes and do not undergo hypertrophy. This characteristic of human iChon cells is favorable when considering their application to cell transplantation for the potential treatment of defects of articular cartilage which consists of hyaline cartilage and seldom undergo hypertrophy. CD-hBMSCs tend to undergo hypertrophy and thus can be lost quickly after cell transplantation [27]. Human iChon cells do not undergo hypertrophy, probably because of their continued expression of the SOX9 transgene.

Cell type conversion through iPS cells is associated with two different risks of tumor formation: one is the risk of teratoma formation associated with the pluripotency of iPS cells [28], and the other is associated with the transduction of the reprogramming factors [13]. The iChon cells are theoretically free from the former risk, because mouse iChon cells do not enter a pluripotent state during induction, as indicated by the lack of Nanog-GFP expression during induction [14]. However, iChon cells are obviously still associated with the latter risk, because c-MYC and KLF4 are used, although the human iChon cells did not produce tumors for at least 3 months after being injected into nude mice. Safer iPS cells have recently been generated by using integration-free vectors such as episomal plasmid vectors [29] and Sendi virus vectors [30]. Because persistent transgene expression is not necessary for the maintenance of mouse iChon cells as long as they are cultured in a chondrogenic medium containing TGF-β and BMPs [12], it will be ideal to generate human iChon cells by transient expression of c-MYC and KLF4 using integration-free vectors, to minimize the risk of tumor formation. The persistent expression of the SOX9 transgene may positively contribute to the stable chondrogenic phenotype of iChon cells without undergoing hypertrophy in this study. If iChon cells are generated by the transient expression of c-MYC, KLF4 and SOX9, such iChon cells would undergo hypertrophy in a manner similar to that of CD-hBMSCs. An engineered cartilage using CD-hBMSCs undergo hypertrophy faster than articular cartilage and thus can be lost quickly after cell transplantation [27]. However, the constitutive expression of Sox9 can be of another concern, because all tissues undergo remodeling *in vivo*, and an engineered cartilage that does not respond to physiological regulation may present long-term challenges. Further study will thus be needed to stringently control the hypertrophy of such iChon cells during cartilage repair.

Human iChon cells differ from mouse induced chondrogenic cells in several aspects. The karyotypes of the majority of human iChon cells were normal, whereas the karyotypes are fairly unstable in mouse iChon cells [12]. Human iChon cell lines did not form tumors when transplanted into nude mice, whereas inappropriately reprogrammed mouse induced chondrogenic cells developed into tumors [12]. A possible reason for the more stable karyotypes and non-tumorigenicity of human iChon cells is related to the limited proliferative activities of human iChon cells, whereas mouse induced chondrogenic cells appear to have almost unlimited proliferative potential [12].

Stable karyotypes and non-tumorigenicity are favorable characteristics of human iChon cells when considering their application to regenerative medicine. Although various obstacles remain, human iChon cells can contribute to the development of cell sources to generate hyaline cartilage-related biomaterials.

## Supporting Information

Figure S1 **The lentiviral** ***COL11A2-*** **reporter vector.** (A) A schematic representation of the lentiviral vectors carrying EGFP-IRES-Puro linked to the *COL11A2* promoter plus the *COL11A2* enhancer. (B) *Left*, EGFP expression in human dermal fibroblasts (HDFs) and human chondrosarcoma (HCS-2/8) cells transduced with the lentiviral *COL11A2-*reporter vector. Bars, 100 µm. *Right*, the results of a flow cytometric analysis of the EGFP expression from the reporter vectors in the cells.
(JPG)

Figure S2 **An immunoblot analysis of the expression of SOX5, SOX6 and SOX9 retroviral vectors in HDF culture.** The Plat-E cells were transfected with pMXs-SOX5, pMXs-SOX6, pMXs-SOX9 and pMXs-EGFP. Supernatants containing each of the retroviruses were added to the HDFs that had been nucleofected with Slc7a1. The cells were lysed 7 days after retroviral transduction, and then were subjected to an immunoblot analysis using anti-SOX5, anti-SOX6 and anti-SOX9 antibodies (Supplementary Table S4) as indicated on the left of membranes (top row). Membranes were reprobed with anti-β-actin antibodies (bottom row).
(JPG)

Figure S3 **The presence of transgenes in iChon cells, karyotypes of iChon cells, and marker gene expression in iChon cells after passage numbers.** **(A)** The presence of transgenes in iChon cells. PCR reactions were performed with template genomic DNA extracted from each iChon cell line using primers specific for each transgene. GAPDH was used as a control. HDF, human dermal fibroblasts. **(B)** The karyotypes of human iChon cells. iChon cell lines #117-3 and #117-37 were examined at passages 18 and 22, respectively. A total of 20 cells for each cell line were examined. **(C)** The results of an analysis of marker gene expression in iChon cell lines (#89-9 and #117-37) after various passage numbers. *P9*, passage 9; *P11*, passage 11; *P13*, passage 13. The expression levels of chondrocyte markers were maintained, and the expression of fibroblast markers was maintained at low levels, after all of the passage numbers examined. Error bars indicate the ± SD (n = 3 dishes). HDFs, human neonatal dermal fibroblasts; HFCs, redifferentiated human fetal chondrocytes.
(JPG)

Figure S4 **The frequencies of prechondrogenic cells in HDF cultures.** (A) Immunofluorescence staining of human dermal fibroblasts (HDF) in one well of a 6 well plate with anti-SOX9 antibodies. The nuclei were stained with PI. Each whole well was scanned as an 8×8 image, and the tiling images were reconstituted using the Biostation CT (Nikon). Top left, a tiling image of SOX9 immunofluorescence. Bottom left, magnification of the boxed region in the top left panel. Phase images (top right) and nuclear stained images with PI (bottom right) corresponding to the bottom left panel. Bars in the top left panels, 10 mm; bars in the bottom left, top right and bottom right panels, 100 µm. (B) As a positive control for SOX9 immunofluorescence, mouse induced chondrogenic MK-7 cells (Hiramatsu, et al., *J Clin Invest* 121(2): 640-57) were used. Bar, 100 µm. (C) The frequencies of cells showing immunoreactivity against anti-Sox9 antibodies in HDF culture. The cell numbers were counted with the CL-Quant software program (Nikon). Three wells of a 6-well plate were analyzed. The positive cell numbers represent the numbers of cells showing immunofluorescence (Alexa Fluor) with anti-Sox9 antibodies.
(JPG)

Figure S5 **Controls for the immunohistochemical analysis.** Histological sections from osteochondroma samples dissected at a time of surgery were immunostained with anti-type I collagen and anti-type II collagen antibodies under the conditions used in this study. The hyaline cartilage of the cartilage cap (arrows) showed immunoreactivity against the anti-type II collagen antibody, but did not show immunoreactivity against the anti-type I collagen antibody. Magnification of boxed regions are shown in Figure 4C. Bar, 1 mm.
(JPG)

Table S1 **The results of the subcutaneous injection of human iChon cell lines into nude mice.**
(DOC)

Table S2 **The sequences of the primers used for the transgenes.**
(DOC)

Table S3 **The sequences of primers for the marker genes, bisulfite sequencing, and PCR cloning.**
(DOC)

Table S4 **The antibodies used for the experiments.**
(DOC)

Movie S1 **Time-lapse images taken during the induction of iChon cells.** HDFs were transduced with the lentiviral *COL11A2* reporter vector, nucleofected with *Slc7a1*, and transduced with retroviral c-MYC, KLF4 and SOX9 vectors. Cells were replated onto a well of a 6 well plate immediately after completion of the retroviral transduction. The well was cultured in the absence of puromycin and subjected to time-lapse GFP observation using the Biostation CT program (Nikon). Phase (Movie S1) and GFP (Movie S2) images were captured every 8 h for 14 consecutive days. Each image is shown for 0.5 sec, thus 8 h corresponds to 0.5 sec.
(AVI)

Movie S2 **Time-lapse images taken during the induction of iChon cells.** HDFs were transduced with the lentiviral *COL11A2* reporter vector, nucleofected with *Slc7a1*, and transduced with retroviral c-MYC, KLF4 and SOX9 vectors. Cells were replated onto a well of a 6 well plate immediately after completion of the retroviral transduction. The well was cultured in the absence of puromycin and subjected to time-lapse GFP observation using the Biostation CT program (Nikon). Phase (Movie S1) and GFP (Movie S2) images were captured every 8 h for 14 consecutive days. Each image is shown for 0.5 sec, thus 8 h corresponds to 0.5 sec.
(AVI)
