# Supplementary figures and images for: Direct Induction of Chondrogenic Cells from Human Dermal Fibroblast Culture by Defined Factors
Source: PLoS One. 2013 Oct 16;8(10):e77365. doi: 10.1371/journal.pone.0077365 (PMC3797820; doi:10.1371/journal.pone.0077365)

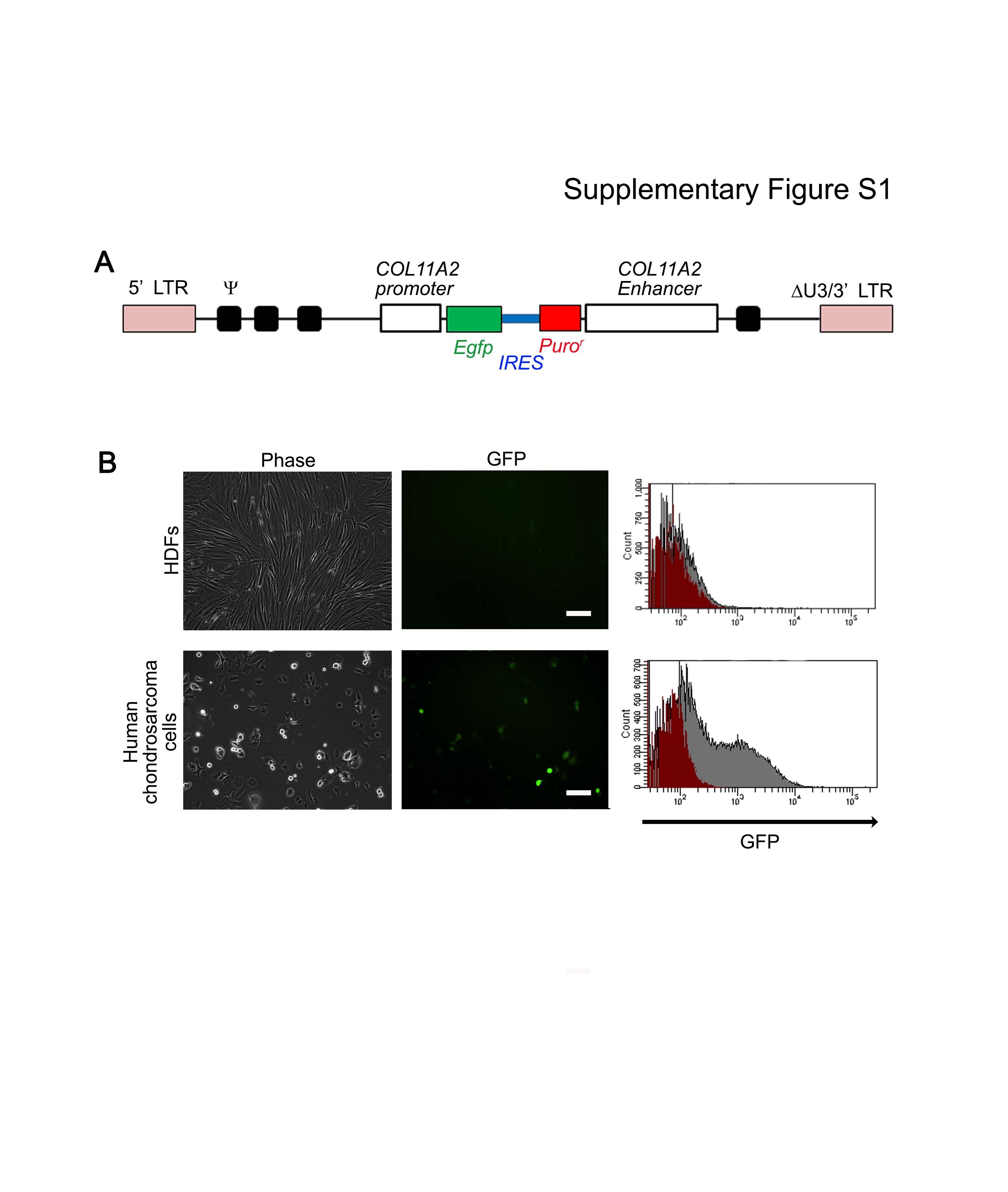

Supplement: Figure S1 — The lentiviral COL11A2- reporter vector. (A) A schematic representation of the lentiviral vectors carrying EGFP-IRES-Puro linked to the COL11A2 promoter plus the COL11A2 enhancer. (B) Left, EGFP expression in human dermal fibroblasts (HDFs) and human chondrosarcoma (HCS-2/8) cells transduced with the lentiviral COL11A2-reporter vector. Bars, 100 µm. Right, the results of a flow cytometric analysis of the EGFP expression from the reporter vectors in the cells. (JPG) [file pone.0077365.s001.jpg]

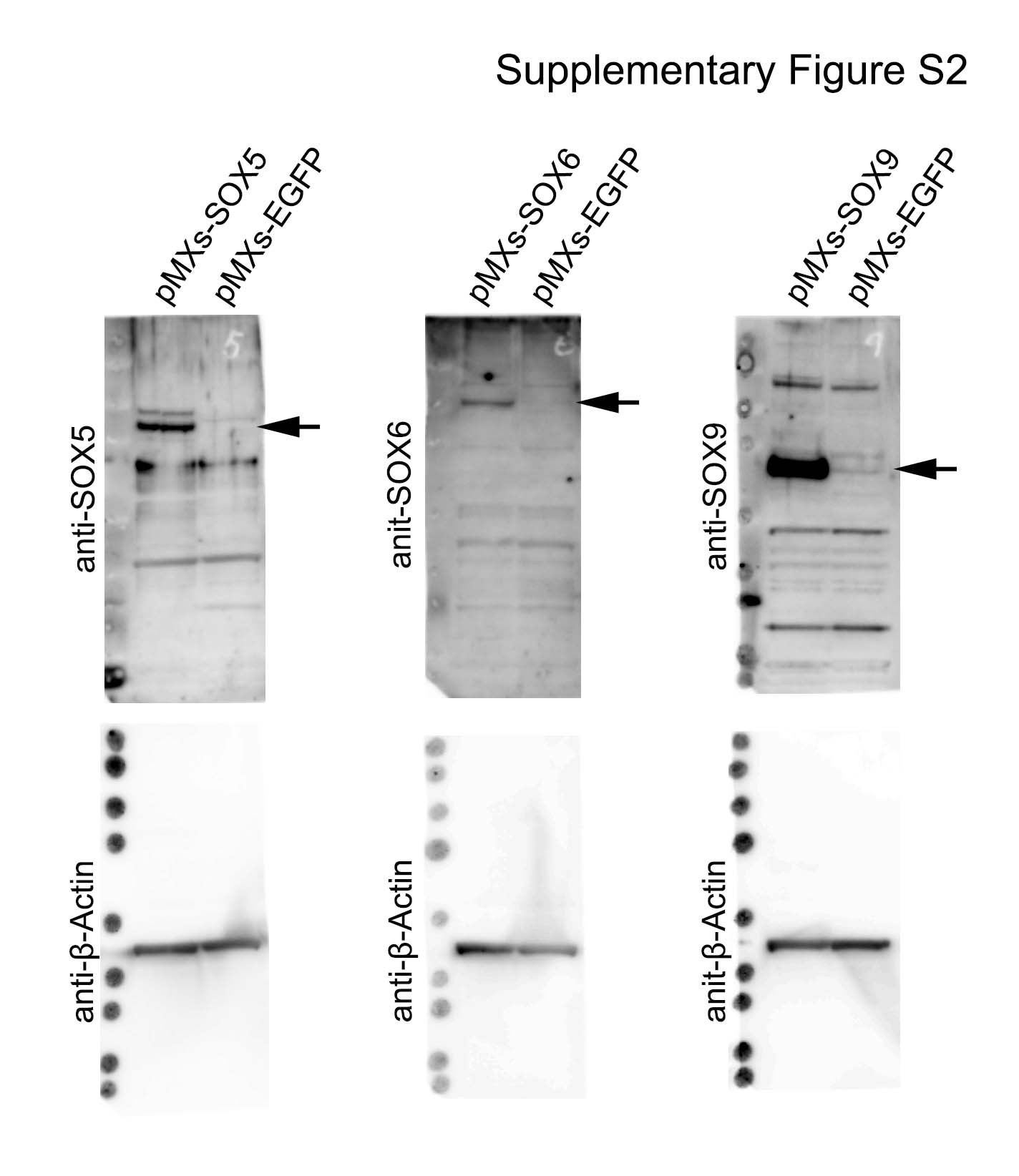

Supplement: Figure S2 — An immunoblot analysis of the expression of SOX5, SOX6 and SOX9 retroviral vectors in HDF culture. The Plat-E cells were transfected with pMXs-SOX5, pMXs-SOX6, pMXs-SOX9 and pMXs-EGFP. Supernatants containing each of the retroviruses were added to the HDFs that had been nucleofected with Slc7a1. The cells were lysed 7 days after retroviral transduction, and then were subjected to an immunoblot analysis using anti-SOX5, anti-SOX6 and anti-SOX9 antibodies (Supplementary Table S4) as indicated on the left of membranes (top row). Membranes were reprobed with anti-β-actin antibodies (bottom row). (JPG) [file pone.0077365.s002.jpg]

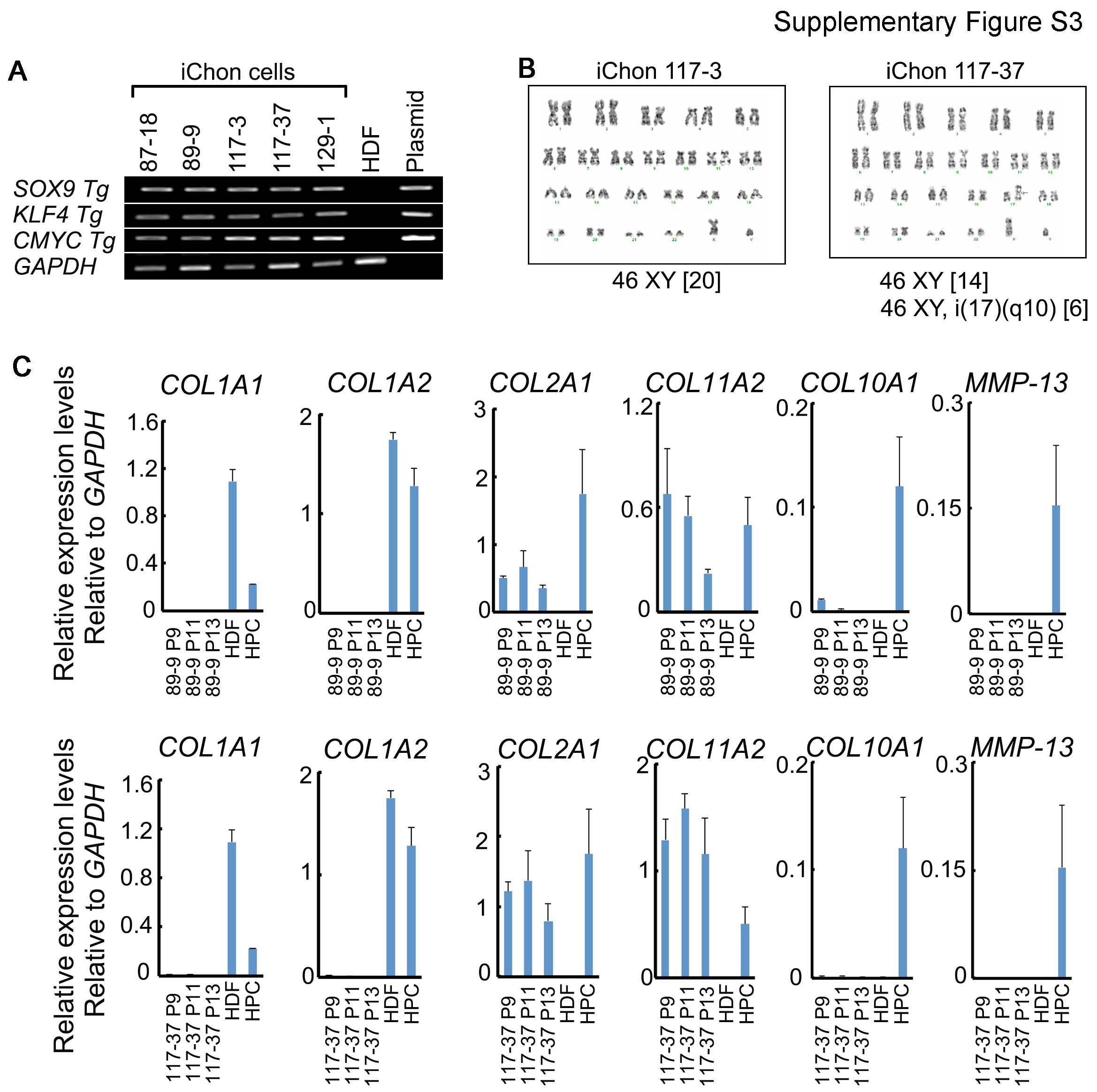

Supplement: Figure S3 — The presence of transgenes in iChon cells, karyotypes of iChon cells, and marker gene expression in iChon cells after passage numbers. (A) The presence of transgenes in iChon cells. PCR reactions were performed with template genomic DNA extracted from each iChon cell line using primers specific for each transgene. GAPDH was used as a control. HDF, human dermal fibroblasts. (B) The karyotypes of human iChon cells. iChon cell lines #117-3 and #117-37 were examined at passages 18 and 22, respectively. A total of 20 cells for each cell line were examined. (C) The results of an analysis of marker gene expression in iChon cell lines (#89-9 and #117-37) after various passage numbers. P9, passage 9; P11, passage 11; P13, passage 13. The expression levels of chondrocyte markers were maintained, and the expression of fibroblast markers was maintained at low levels, after all of the passage numbers examined. Error bars indicate the ± SD (n = 3 dishes). HDFs, human neonatal dermal fibroblasts; HFCs, redifferentiated human fetal chondrocytes. (JPG) [file pone.0077365.s003.jpg]

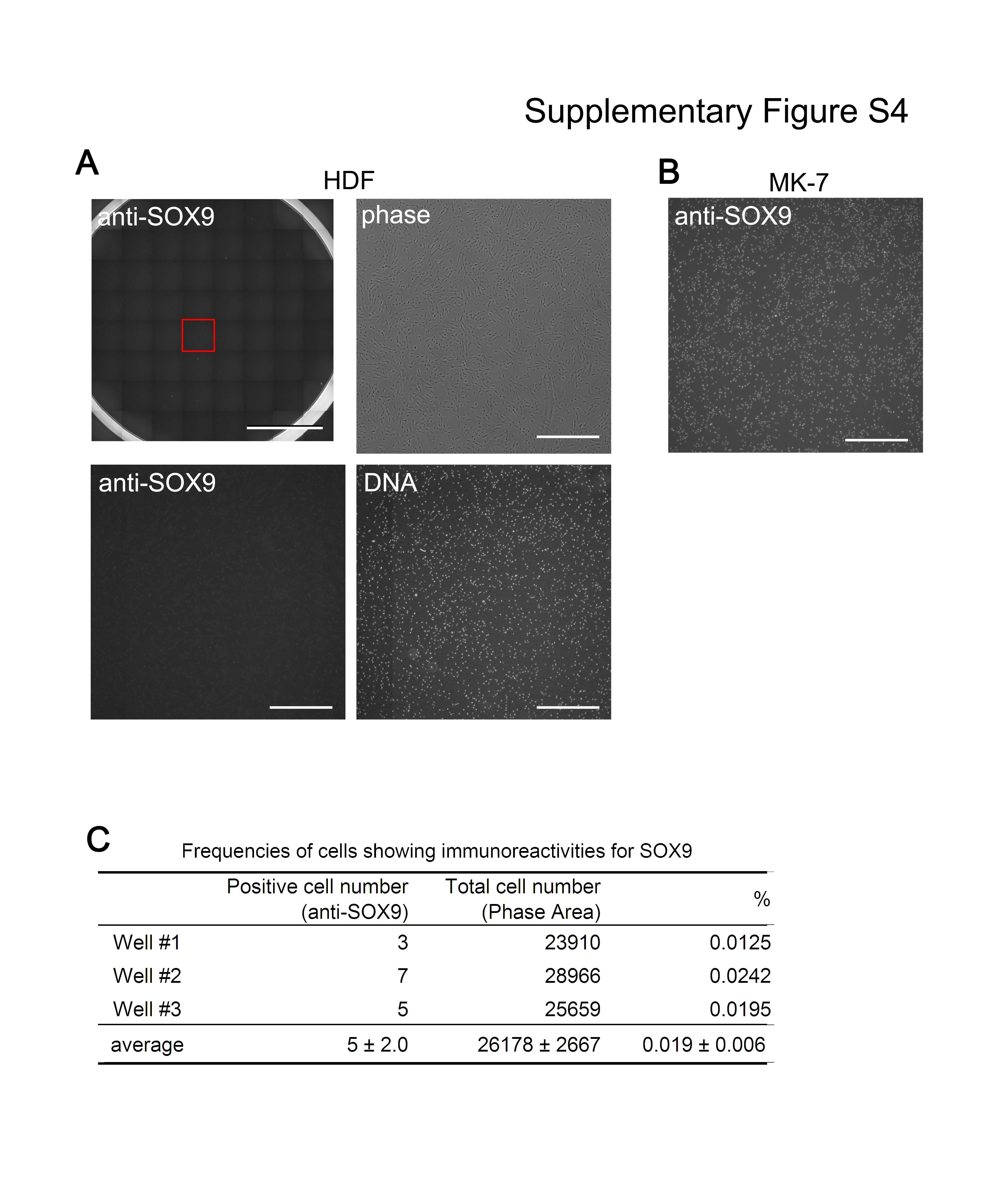

Supplement: Figure S4 — The frequencies of prechondrogenic cells in HDF cultures. (A) Immunofluorescence staining of human dermal fibroblasts (HDF) in one well of a 6 well plate with anti-SOX9 antibodies. The nuclei were stained with PI. Each whole well was scanned as an 8×8 image, and the tiling images were reconstituted using the Biostation CT (Nikon). Top left, a tiling image of SOX9 immunofluorescence. Bottom left, magnification of the boxed region in the top left panel. Phase images (top right) and nuclear stained images with PI (bottom right) corresponding to the bottom left panel. Bars in the top left panels, 10 mm; bars in the bottom left, top right and bottom right panels, 100 µm. (B) As a positive control for SOX9 immunofluorescence, mouse induced chondrogenic MK-7 cells (Hiramatsu, et al., J Clin Invest 121(2): 640-57) were used. Bar, 100 µm. (C) The frequencies of cells showing immunoreactivity against anti-Sox9 antibodies in HDF culture. The cell numbers were counted with the CL-Quant software program (Nikon). Three wells of a 6-well plate were analyzed. The positive cell numbers represent the numbers of cells showing immunofluorescence (Alexa Fluor) with anti-Sox9 antibodies. (JPG) [file pone.0077365.s004.jpg]

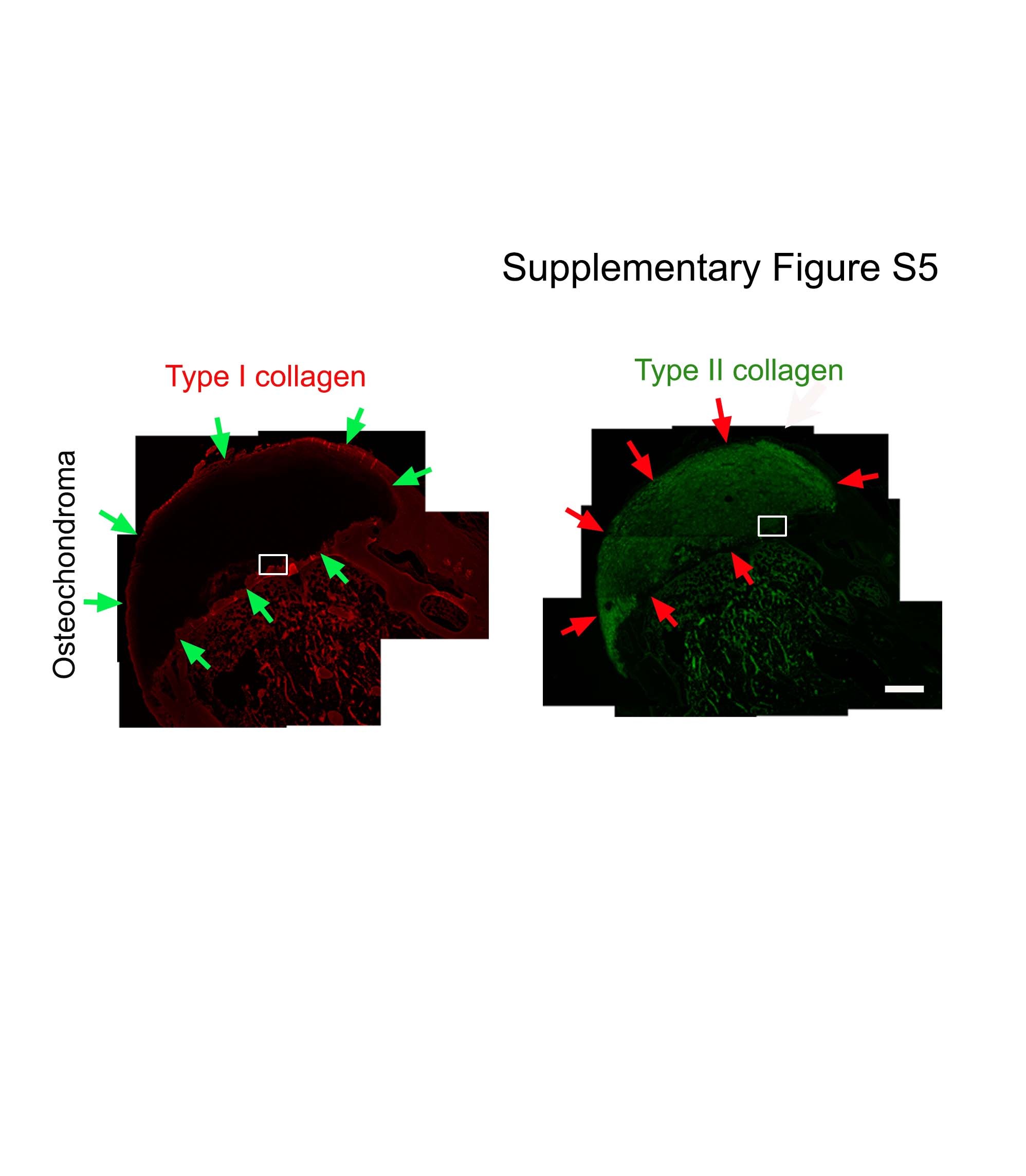

Supplement: Figure S5 — Controls for the immunohistochemical analysis. Histological sections from osteochondroma samples dissected at a time of surgery were immunostained with anti-type I collagen and anti-type II collagen antibodies under the conditions used in this study. The hyaline cartilage of the cartilage cap (arrows) showed immunoreactivity against the anti-type II collagen antibody, but did not show immunoreactivity against the anti-type I collagen antibody. Magnification of boxed regions are shown in Figure 4C. Bar, 1 mm. (JPG) [file pone.0077365.s005.jpg]
