# Supplementary material for: Direct Induction of Chondrogenic Cells from Human Dermal Fibroblast Culture by Defined Factors
Source: PLoS One. 2013 Oct 16;8(10):e77365. doi: 10.1371/journal.pone.0077365 (PMC3797820; doi:10.1371/journal.pone.0077365)
Supplement: Table S1 — The results of the subcutaneous injection of human iChon cell lines into nude mice. (DOC) [file pone.0077365.s006.doc]

Supplementary Table S1. The results of the subcutaneous injection of human iChon cell lines into nude mice.

| Time | Clone | Site | Cartilage | Tumor |
| --- | --- | --- | --- | --- |
| 4W | 87-18 | 4 | 3 | 0 |
|  | 89-9 | 4 | 0 | 0 |
|  | 117-3 | 12 | 9 | 0 |
|  | 117-8 | 4 | 1 | 0 |
|  | 117-37 | 14 | 0 | 0 |
|  | 117-38 | 4 | 1 | 0 |
| 8W | 87-18 | 3 | 1 | 0 |
|  | 89-9 | 4 | 0 | 0 |
|  | 117-3 | 2 | 1 | 0 |
|  | 117-8 | 2 | 0 | 0 |
|  | 117-37 | 4 | 0 | 0 |
|  | 117-38 | 2 | 0 | 0 |
| 12W | 87-18 | 2 | 0 | 0 |
|  | 89-9 | 2 | 0 | 0 |
|  | 117-3 | 2 | 2 | 0 |
|  | 117-37 | 2 | 0 | 0 |
|  | 138-6 | 4 | 0 | 0 |
|  | 138-20 | 1 | 1 | 0 |
|  | 138-25 | 2 | 0 | 0 |
|  | 138-37 | 1 | 0 | 0 |
|  | 138-39 | 1 | 0 | 0 |
