# Supplementary material for: Direct Induction of Chondrogenic Cells from Human Dermal Fibroblast Culture by Defined Factors
Source: PLoS One. 2013 Oct 16;8(10):e77365. doi: 10.1371/journal.pone.0077365 (PMC3797820; doi:10.1371/journal.pone.0077365)
Supplement: Table S2 — The sequences of the primers used for the transgenes. (DOC) [file pone.0077365.s007.doc]

Supplementary Table S2. The sequences of the primers used for the transgenes

| Primer | | Sequence |
| --- | --- | --- |
| Genomic PCR analysis | | |
| *GAPDH* RT S | | ACCCAGAAGACTGTGGATGG |
| *GAPDH* RT AS | | TTCTAGACGGCAGGTCAGGT |
| *SOX9* Tg RT S | | GTGGTGGTACGGGAAATCAC |
| *SOX9* Tg RT AS | | CGCTCTCCTTCTTCAGATCG |
| *c-MYC* Tg RT S | | GTGGTGGTACGGGAAATCAC |
| *c-MYC* Tg RT AS | | AGCAGCTCGAATTTCTTCCA |
| *KLF4* Tg RT S | | GACCACCTCGCCTTACACAT |
| *KLF4* Tg RT AS | | TTTCCTTACGCGAAATACGG |
| Transgene expression analysis | | |
| *SOX9* RT end + tg S | ACCACCAGAACTCCAGCTC | |
| *SOX9* RT end + tg AS | CACTGTGCTGGATATCAGACC | |
| *SOX9* RT end S | gatggccgagatgatcctaa | |
| *SOX9* RT end AS | tgctccatttagccaaggtt | |
| *KLF4* RT end + tg S | GACCGCCACCCACACTTGTGATTA | |
| *KLF4* RT end + tg AS | TGCTCGGTCGCATTTTTGGCAC | |
| *KLF4* RT end S | tatgacccacactgccagaa | |
| *KLF4* RT end AS | atccagtcacagaccccatc | |
| *c-MYC* RT end + tg S | tcctcggattctctgctctc | |
| *c-MYC* RT end + tg AS | ctctgaccttttgccaggag | |
| *c-MYC* RT end S | cgattccttctaacagaaatgtcc | |
| *c-MYC* RT end AS | tcttttatgcccaaagtcca | |
