# Supplementary material for: Direct Induction of Chondrogenic Cells from Human Dermal Fibroblast Culture by Defined Factors
Source: PLoS One. 2013 Oct 16;8(10):e77365. doi: 10.1371/journal.pone.0077365 (PMC3797820; doi:10.1371/journal.pone.0077365)
Supplement: Table S3 — The sequences of primers for the marker genes, bisulfite sequencing, and PCR cloning. (DOC) [file pone.0077365.s008.doc]

Supplementary Table S3. The sequences of primers for the marker genes, bisulfite sequencing, and PCR cloning.

| Primer | Sequence |
| --- | --- |
| Marker gene expression analysis | |
| *GAPDH* RT S | ACCCAGAAGACTGTGGATGG |
| *GAPDH* RT AS | TTCTAGACGGCAGGTCAGGT |
| *COL2A1* RT S | GTGGAGCAGCAAGAGCAA |
| *COL2A1* RT AS | TGTTGGGAGCCAGATTGT |
| *COL10A1* RT S | ATGCTGCCACAAATACCCTTT |
| *COL10A1* RT AS | GGAATGAAGAACTGTGTCTTGGT |
| *MMP13* RT S | ACTGAGAGGCTCCGAGAAATG |
| *MMP13* RT AS | TGTTATCGTCAAGTTTGCCAGTC |
| *COL1A1* RT S | GTCGAGGGCCAAGACGAAG |
| *COL1A1* RT AS | CAGATCACGTCATCGCACAAC |
| *COL1A2* RT S | AATTGGAGCTGTTGGTAACGC |
| *COL1A2* RT AS | CACCAGTAAGGCCGTTTGC |
| *ACAN* RT S | AGGAGACAGAGGGACACGTC |
| *ACAN* RT AS | TCCACTGGTAGTCTTGGGCAT |
| *BGLAP RT F* | CACTCCTCGCCCTATTGGC |
| *BGLAP RT R* | CCCTCCTGCTTGGACACAAAG |
| *ALPL F* | tgtaaggacatcgcctacc |
| *ALPL R* | cgtcactctcatactccaca |
| *SP7 OSTERIX F* | acctcaggctatgctaatga |
| *SP7 OSTERIX R* | gtagacactgggcagacagt |
| *RUNX2 RT F* | accatggtggagatcatcg |
| *RUNX2 RT R* | cgccatgacagtaaccacag |
| Bisulfite genomic sequencing | |
| *COL1A1* BS S | GGGAGTAGTATTAGTAAATTTTGGTTTTAA |
| *COL1A1* BS AS | AACCATACCCACCTACAACCC |
| PCR cloning |  |
| SOX5 F attB1 | ggggacaagtttgtacaaaaaagcaggcttcgaaggagatagaaccatgtcttccaagcgaccagcct |
| SOX5 R attB2 | ggggaccactttgtacaagaaagctgggtctcagttggcttgtcctgcaatatgg |
| SOX6 F attB1 | ggggacaagtttgtacaaaaaagcaggcttcgaaggagatagaaccatgtcttccaagcaagccacctc |
| SOX6 R attB2 | ggggaccactttgtacaagaaagctgggtctcagttggcactgacagcctc |
