# Supplementary material for: Direct Induction of Chondrogenic Cells from Human Dermal Fibroblast Culture by Defined Factors
Source: PLoS One. 2013 Oct 16;8(10):e77365. doi: 10.1371/journal.pone.0077365 (PMC3797820; doi:10.1371/journal.pone.0077365)
Supplement: Table S4 — The antibodies used for the experiments. (DOC) [file pone.0077365.s009.doc]

Supplementary Table S4. The antibodies used for the experiments

| Antibody | Source (Cat. No). | Dilution |
| --- | --- | --- |
| Anti-type I collagen (mouse monoclonal) | Abcam (ab6308) | 1/100 |
| Anti-type II collagen (mouse monoclonal) | Thermo (#MS-235-P0) | 1/200 |
| Anti-aggrecan (rabbit polyclonal) | Santa Cruz (sc-25674) | 1/200 |
| Anti-SOX9 (rabbit polyclonal) | Santa Cruz (sc-20095) | 1/200 |
| Anti-type X collagen (mouse monoclonal) | Quartett (2031501001) | 1/200 |
| Anti-human Vimentin (rabbit monoclonal) | Abcam (ab16700) | 1/100 |
| Alexa Fluor 488 mouse anti-rabbit | Invitrogen (A11001) | 1/2000 |
| Alexa Fluor 488 rabbit anti-mouse | Invitrogen (A21206) | 1/2000 |
| Anti-SOX5 (rabbit polyclonal) | Santa Cruz (sc20091) | 1/200 |
| Anti-SOX6 (goat polyclonal) | Santa Cruz (sc17332) | 1/200 |
| Anti-b-actin (rabbit polyclonal) | cell signaling (#4967) | 1/1000 |
| Anti-rabbit IgG, HRP-linked | GE healthcare (NA934) | 1/2000 |
| Anti-goat IgG-HRP | Santa Cruz (sc-2020) | 1/5000 |
